# NOX enzymes as drug targets

**DOI:** 10.1007/s00018-012-1006-5

**Published:** 2012-05-15

**Authors:** Karl-Heinz Krause, David Lambeth, Martin Krönke

**Affiliations:** 1 Department of Pathology and Immunology, Geneva Medical Faculty, Centre Medical Universitaire, 1, rue Michel-Servet, 1211 Geneva, Switzerland; 2 Department of Genetic and Laboratory Medicine, Geneva University Hospitals, Centre Medical Universitaire, 1, rue Michel-Servet, 1211 Geneva, Switzerland; 3 Department of Pathology and Laboratory Medicine, University Medical School-Emory University, Room #148, Whitehead Research Building, 1510 Clifton Road, Atlanta, GA 30322 USA; 4 Institut für Medizinische Mikrobiologie, Immunologie und Hygiene, Universität zu Köln, Goldenfelsstr 19–21, 50935 Cologne, Germany; 5 Center for Molecular Medicine Cologne, CECAD Cologne and German Center of Infection Research, Cologne, Germany

**Keywords:** NADPH oxidases (NOX), Peptide inhibitors, Reactive oxygen species (ROS), NOX and disease

The Nox field (for review see [3, 4] has seen two distinct epochs: (1) the Age of the Phagocyte Oxidase, which began in earnest more than 30 years ago, during which the respiratory burst oxidase was believed to be the sole “professional” generator of reactive oxygen species (ROS), the production of which was thought to be largely confined to phagocytic cells, and (2) the Age of the Nox Family, which began around the turn of the 21st century and which showed the existence of multiple Nox isoenzymes. The Age of the Phagocyte Oxidase revealed that an NADPH-dependent, superoxide-generating respiratory burst oxidase is essential for microbial killing by phagocytes, elucidated the key catalytic (Nox2) and regulatory (p22*phox*, p47*phox*, p67*phox*, p40*phox*, Rac) subunits that control this enzyme, and showed that the genetic absence or mutation of individual NADPH oxidase components causes chronic granulomatous disease wherein affected individuals are prone to frequent, unusual infections. During the subsequent Age of the Nox Family, it was discovered that there are multiple Nox isoenzymes (seven in human—Nox1, Nox2, Nox3, Nox4, Nox5, Duox1, and Duox2—plus many more that are widely distributed among virtually all eukaryotic organisms from fungus to vertebrates), that they exist in a variety of cell types/tissues, and that they show varied mechanisms of activation. Mostly during the 1990s (prior to and leading up to the second Nox epoch) investigators in diverse fields had observed that superoxide or hydrogen peroxide was produced in their non-phagocyte tissue/cell *de jour*, often in response to a hormone or growth factor. Nevertheless, the phagocyte oxidase was usually absent, sometimes leading to the misidentification of the source of ROS as mitochondria or a side reaction by another enzyme. In addition, the 1990s gave rise to early clues that ROS might be important in the pathogenesis of disease states in a range of tissues. Thus, by the early 2000s, many clinically important fields were poised to make the most of the newly discovered Nox family. This has resulted in a decade of enormous cross-disciplinary excitement and growth, and has required those of us who grew up in the first Nox epoch to become fluent in a variety of new clinical “languages”.

In the journey from lab bench to bedside, there are a series of predictable stages through which a scientific discovery—in this case a group of enzymes—evolves. In the first stage, the basic science is extended in an effort to understand the fundamental enzymatic or regulatory mechanisms and protein or ligand interactions. Among other important findings for Nox enzymes, this still-ongoing phase has yielded information as to the different modes of regulation (the new regulatory subunits NOXO1 and NOXA1, calcium, phosphorylation, controlled expression, oligomerization, etc.) and structural features (e.g*.*, binding sites, regulatory domains, etc.) that are important to the function of Nox enzymes. Such information provides the foundation for all subsequent stages, including designing methods to screen for novel inhibitors and characterizing how these inhibitors work. A second stage attempts to answer the question: “What are the normal biological roles of the Nox isoform(s)”? In the early 2000s, the new Nox isoenzymes were somewhat enigmatic in this regard, due to preconceptions about “universally harmful effects” of ROS, along with biases from the phagocyte oxidase that predisposed investigators to interpret their results in terms of innate immune mechanisms. This second stage catalogs when and where the enzymes are expressed and/or activated, and relies heavily on methods to suppress the expression of a given Nox isoform in cells or animals. The latter approach has been particularly valuable in establishing biological roles for Nox isoforms above and beyond microbial killing; these include chemical modification/stabilization of extracellular matrix (*C. elegans*), signal transduction in smooth muscle (*Drosophila*), gravity perception (mouse), etc. A third evolutionary stage attempts to define a role for the enzyme/protein in disease states. This stage historically can lag the initial discovery by many years or decades (if it occurs at all!). However, in the case of Nox enzymes, investigators in many clinically important fields had already suspected ROS in pathogenesis, and had been on the lookout for the enzyme perpetrator(s). Following the identification of the Nox Family, suspicion quickly fell on the Nox isozymes and in some cases specific Noxes have been pronounced guilty, based to a large extent upon animal models of disease. The fourth stage, after a given enzyme has been incriminated, is to utilize fundamental information about the enzyme to discover ways to intervene therapeutically. This usually means discovering an inhibitor that blocks function, but in some cases can involve suppression of the enzyme’s expression, e.g., using RNA interference or other genetic means. When successful, the outcome is a drug or other therapeutic method [1, 2]. Given the short history of the Age of the Nox Family, there is so far no fully developed Nox isoform-based therapeutic method. However, several academic laboratories and/or small pharmaceutical companies are dedicated to this approach and have developed Nox inhibitors that show selectivity for different Nox isoenzymes. Indeed, we are aware of one such inhibitor that is already in phase I clinical trials for diabetic nephropathy, and more drug candidates will undoubtedly follow.

This volume summarizes the remarkable progress that now implicates individual Nox isoforms in a surprising number of specific, mostly chronic, diseases. While the Nox enzymes themselves are unlikely to be the precipitating event, sufficient evidence now exists to accuse them and their gang of ROS of being key offenders in causing cell and tissue damage in diverse conditions. This volume reflects this growing appreciation that Nox isoforms participate in many diseases, and an expanding interest in developing Nox-based therapeutics. 

With an obvious predilection for quotations from Winston Churchill, the first contribution by Iris Dahan and Edgar Pick, Tel Aviv University, starts with an introduction into the family of NADPH oxidases followed by the rational for the identification and development of Nox inhibitors. The authors briefly dip into the history of Nox inhibitory peptides and then deal with the challenges, opportunities, and pitfalls of peptide inhibitors in general. This comprehensive and thoughtful review is a scientific as well as literary highlight that sets the stage for the following contributions.

Jamel El-Benna and coworkers, INSERM, Paris, focus on peptide inhibitors specific for the phagocyte NADPH oxidase, Nox2 (previously called gp91phox). The mode of action is discussed with special emphasis on possible targets including either Nox2 itself and/or molecules of the Nox2 activation complex. Furthermore, the challenge of delivery into living cells is discussed. One option suggested is the use of cell-penetrating peptides or protein-transduction domains identified in the HIV TAT protein or the antennapedia protein from *Drosophila*. Eventually, the beneficial potency of Nox2 inhibitors in inflammatory diseases is highlighted.

The theme of Nox inhibitory peptides is taken up by the Pagano group, University of Pittsburgh, who start out with the notion that any of the available inhibitors have proven non-specific, falling into the category of scavengers or inhibitors of more than one source of ROS. They review some of the efforts that have been undertaken to develop specific inhibitors of Nox oxidases over the past decade. Nox inhibitory peptides such as Nox2ds-tat were valued for their specificity. However, the “druggability” of peptides is generally challenged due to their limited bioavailability, gut degradation, and inability to cross the plasma membrane of living cells. Given the limitations of peptide-inhibitor strategies, the search for specific Nox inhibitors has returned to small-molecule inhibitors by using high-throughput screening of diverse libraries. Pagano et al*.* discuss a number of compounds recently identified by HTS to come to the conclusion that only one small molecule, ML171, thus far proved specific for one Nox isoform (Nox1). The development of isoform-specific Nox inhibitors is considered mandatory for curtailing their many in vivo effects, which is required for therapeutical applicability. Given the high degree of homology among the various Nox isoforms in terms of catalytic activity and structure, the identification of isoform-specific Nox inhibitors seems to be a formidable task.

One of these small molecule inhibitors, VAS2870, is one topic of the contribution by Harald Schmidt and coworkers, Maastricht University. The review of the literature characterized VAS2870 as a pan-Nox inhibitor that blocks the activity of Nox1,-2, and -4 as well as Duox (in zebrafish). Although the mode of action is obviously non-specific for Nox isoforms, VAS2870 is processed further for preclinical testing. In a second part, this work dwells on Nox4 as a possible therapeutic target. Based on the observation that Nox4 knock-out mice do not show an overt phenotype, the authors first suggest that Nox4 inhibition would probably not cause severe complications. They then dialectically discuss the potential clinical outcome of Nox4 inhibition in consideration of the protective roles of Nox4. They conclude that acute ischemic stroke appears to be one of the most promising and safest indications for Nox4 inhibition, because prolonged Nox4 inhibition as therapeutic modality for chronic diseases may compromise the protective role of Nox4 in heart failure and angiogenesis.

The contribution of Timo Kahles and Ralph Brandes focuses on reactive oxygen species and NOX enzymes in ischemic brain injury. The authors point out the apparent contradiction between the well-established role of ROS in experimental models of ischemic stroke on one hand, and the inefficacy of antioxidants on the other hand. The authors conclude that a clinical translation of the oxidative stress concept in cerebrovascular disease “demands advanced approaches like targeting the source of ROS generation, not their products.” The authors then discuss the role of ROS in the breakdown of the blood–brain barrier during ischemia reperfusion injury. They provide a review on NOX NADPH oxidases in the cerebral vasculature and summarize our present knowledge of the role of different Nox isoforms. They finally review ischemic stroke experiments in NOX-deficient mice as well as data on stroke therapy with compounds targeting NOX NADPH oxidases. They conclude that NOX-targeted therapies are of major interest for future stroke research, but point out the requirement to develop inhibitors targeting specific NOX isoforms.

The contribution of Victor Thannickal and colleagues focuses on the question of whether NOX inhibitors might provide a therapeutic avenue for pulmonary fibrosis. The authors point out that ROS may have very distinct effects on different cell types. For example, in pulmonary epithelial cells, ROS may lead to cell death, and in contrast, in ROS, lead to an alteration of cell phenotype and resistance to apoptosis. Thus, ROS might be involved in two key aspects of pulmonary fibrosis: epithelial cell apoptosis and the increase in fibroblasts, in particular myofibroblasts. The authors discuss that despite the complexity of pulmonary fibrosis, NOX4 appears to be the predominant source of ROS in the disease. Yet, there might be a contribution of NOX2 coming from inflammatory cells. There is some indication for an activity of* N*-acetyl cysteine in pulmonary fibrosis, however the authors suggest that NOX4 inhibitors are the most promising avenue.

The review by Stephanie Carnesecchi and colleagues focuses on acute lung injury and ARDS (adult respiratory distress syndrome). The group had previously demonstrated that, in a mouse model, NOX1 in alveolar epithelial cells plays an important role in the mediation of hyperoxic lung damage. Yet, based on a review of the available literature, they conclude that in ARDS and acute lung injury, at least three Nox enzymes are involved: NOX1, NOX2, and NOX4. Both NOX1 and NOX4 might contribute to epithelial cell death. NOX4, in addition, however, is likely to also be involved in fibroblast proliferation and fibrotic responses. Finally, NOX2 is probably most important in ARDS-associated inflammatory responses. Thus, it is possible that large-spectrum Nox inhibitors might be most efficient in acute lung injury and ARDS.

The review by Silvia Sorce and colleagues provides an overview of the opportunities for NOX inhibitors to treat diseases of the central nervous system. The authors discuss the role of NOX overactivity in a variety of CNS diseases, from amyotrophic lateral sclerosis to schizophrenia. They also point out that in autoimmune diseases of the central nervous system, insufficient Nox activity might be the cause of an overshooting immune response. They then give an overview of compounds that could be used to target ROS and NOX enzymes in the CNS. They summarize experience with antioxidants, natural compounds, as well as chemically synthesized small molecules. They finally discuss the hurdles that need to be overcome before NOX inhibition can become clinical reality.

The review of Michael Surace and Michelle Block focuses on microglia-mediated neurotoxicity. Microglial cells are professional phagocytes of the central nervous system and hence important cells involved in the host defense and probably also in the removal of unwanted material. Yet, an excessive activity of microglia may also lead to damage of the surrounding brain cells, with NOX2-derived ROS being one of the key neurotoxic mechanisms. The authors focus particularly on the role of NOX2 and microglia in Alzheimer’s disease and in Parkinson’s disease. Both pathologies are characterized by a marked neuroinflammation, including microglia activation. The authors describe the experimental impact of available compounds with NOX inhibitory activity, and finish with the description of the potential of specific NOX inhibitors for neuroinflammatory diseases and neurodegeneration.

The review of Rybak and colleagues focuses on the ROS-mediated damage of the inner ear as a cause of hearing loss. More specifically, the authors describe experiments performed with the chemotherapeutic agent cisplatin, a clinically important drug whose usefulness is limited by its ototoxicity. The authors have previously demonstrated that cisplatin-mediated ototoxicity is due to NOX3-dependent ROS generation. Importantly, trans-tympanic injection of siRNA directed against NOX3 protects against cisplatin-induced hearing loss in animal models. The authors conclude that NOX3 inhibition might be a promising way to protect the inner ear from insult, not only from cisplatin, but also from other ototoxic compounds, and even from noise- and age-induced damage.

Michael Bonner and Jack Arbiser finally discuss which inflammatory and malignant disorders can be targeted by NOX inhibitors. This review elucidates the mechanisms of carcinogenetic and inflammatory action of reactive oxygen. These authors emphasize the need to identify ROS-driven phenotypes in specific cancer entities and inflammatory diseases, because pathologic conditions that are characterized by excessive ROS production will likely be responsive to ROS inhibitors, while conditions with defective ROS production may be responsive to ROS inducers.

In summary, there is an emerging new field of pharmacology, namely NOX inhibitors. A wide spectrum of compounds, from large spectrum NOX inhibitors to isoform-specific inhibitors is likely to emerge. As NOX-derived ROS are important disease modifiers in many pathologies, NOX inhibitors might become clinically important drugs in the future.
